# Serum klotho is inversely associated with metabolic syndrome in chronic kidney disease: results from the KNOW-CKD study

**DOI:** 10.1186/s12882-019-1297-y

**Published:** 2019-04-03

**Authors:** Hyo Jin Kim, Joongyub Lee, Dong-Wan Chae, Kyu-Beck Lee, Su Ah Sung, Tae-Hyun Yoo, Seung Hyeok Han, Curie Ahn, Kook-Hwan Oh

**Affiliations:** 1 Department of Internal Medicine, Dongguk University College of Medicine, Gyeongju-si, Gyeongsangbuk-do Korea; 2 School of Medicine, Inha University, Incheon, Korea; 3 Department of Prevention and Management, Inha University Hospital, Incheon, Korea; 4 Department of Internal Medicine, Seoul National University Bundang Hospital, Seongnamsi, Gyeonggi-do Korea; 5 Department of Internal Medicine, Kangbuk Samsung Hospital, Sungkyunkwan University School of Medicine, Seoul, Korea; 6 Department of Internal Medicine, Nowon Eulji Medical Center, Eulji University, Seoul, Korea; 7 Department of Internal Medicine, Yonsei University College of Medicine, Seoul, Korea; 8 Department of Internal Medicine, Seoul National University Hospital, Seoul National University College of Medicine, 101 Daehak-Ro, Jongno-Gu, Seoul, 03080 Korea

**Keywords:** Chronic kidney disease, Klotho, Metabolic syndrome

## Abstract

**Background:**

Metabolic syndrome (MS) is prevalent in chronic kidney disease (CKD). Klotho, a protein linked to aging, is closely associated with CKD. Each component of MS and klotho has an association. However, little is known about the association between klotho and MS per se*.* We investigated the association between serum klotho levels and MS using baseline cross-sectional data obtained from a large Korean CKD cohort.

**Methods:**

Of the 2238 subjects recruited in the KoreaN Cohort Study for Outcome in Patients With Chronic Kidney Disease (KNOW-CKD) between 2011 and 2016, 484 patients with missing data on serum klotho and extreme klotho values (values lower than the detectable range or > 6000 pg/mL) or with autosomal dominant polycystic kidney disease patients were excluded. The data of the remaining 1754 subjects were included in the present study. MS was defined using the revised National Cholesterol Education Program Adult Treatment Panel (NCEP-ATP) III criteria. Serum klotho levels were measured using an enzyme-linked immunosorbent assay.

**Results:**

Mean patient age was 54.9 ± 12.1 years and 1110 (63.3%) were male. The prevalence of MS among all study subjects was 63.7% (*n* = 1118). The median serum klotho level was 527 pg/mL (interquartile range [IQR]: 418–656 pg/mL). Serum klotho level was significantly lower in MS patients than patients without MS (Median [IQR]; 521 pg/mL [413, 651] vs. 541 pg/mL [427, 676], respectively; *P* = 0.012). After adjusting for age, sex, estimated glomerular filtration rate, and overt proteinuria, serum klotho was independently associated with MS (adjusted odds ratio [OR], 0.44; 95% confidence interval, 0.23–0.82; *P* = 0.010). Furthermore, the adjusted OR for MS was found to be significantly increased at serum klotho levels of < 518 pg/mL (receiver operating characteristic curve cut-off value).

**Conclusions:**

Serum klotho was inversely associated with the presence of MS in patients with CKD.

**Trial registration:**

This trial was registered on ClinicalTrials.gov on 26 June 2012 (https://clinicaltrials.gov;NCT01630486).

## Background

Metabolic syndrome (MS) is a cluster of risk factors for diabetes and cardiovascular (CV) disease and is characterized by hypertension, hyperglycemia, hypertriglyceridemia, decreased high-density lipoprotein (HDL) cholesterol, and abdominal obesity [1, 2]. Furthermore, MS is common in chronic kidney disease (CKD) patients [3]. Previous studies showed that MS was independently associated with an increased risk for CKD and vice versa [4, 5].

Klotho, a protein linked to aging, is closely associated with CKD. In a previous study, klotho knock-out mice exhibited similarities with CKD patients, such as, hyperphosphatemia, ectopic soft tissue calcification, and arteriosclerosis [6], which suggested CKD might result from a state of klotho deficiency. Thus, in addition to serving as a biomarker for CKD, klotho deficiency is also viewed as a pathogenetic indicator of renal and extra-renal complications in CKD [7]. Recent reports indicated klotho is associated with each component of MS. Arking et al. [8] showed that a functional variant of the *Klotho* gene was associated with increased systolic blood pressure and decreased HDL-cholesterol levels in 525 Jewish subjects. In experimental studies, klotho has been shown to ameliorate vascular endothelial dysfunction, to increase nitric oxide production, and to reduce elevated blood pressure in an animal models of MS [9, 10]. *Klotho* polymorphism was significantly associated with glucose metabolism in apparently healthy Korean females [11]. In addition, genetic variants of *Klotho* have been associated with insulin resistance and hypertriglyceridemia [12]. Accordingly, it was suggested in all of these studies that klotho be considered a candidate molecule in MS. However, little is known about the association between klotho and MS per se in CKD patients. In the present study, we investigated the metabolic profile characteristics of Korean CKD patients and evaluated the association between serum klotho and MS using baseline cross-sectional data obtained from a large Korean CKD cohort.

## Methods

### Study population

We conducted a cross-sectional analysis of clinical profiles obtained at enrollment for the KoreaN Cohort Study for Outcome in Patients With Chronic Kidney Disease (KNOW-CKD) study. KNOW-CKD is a nationwide prospective cohort study, including predialysis subjects with CKD from stage 1 to 5, aged between 20 and 75 years and recruited from nine clinical centers of major university-affiliated Korean hospitals. Details of the rationale and study design of the KNOW-CKD have been described elsewhere [13]. Of 2238 CKD patients recruited between June 2011 and January 2016, 484 were excluded for the following reasons: 125 for missing serum klotho data; 9 for a serum klotho level lower than the detectable range; 3 for an extremely higher klotho level (> 6000 pg/mL); and 347 for autosomal dominant polycystic kidney disease patients because large cystic masses in their kidneys and liver can cause an overestimation of abdominal obesity. Extreme klotho values were not influence value by Cook’s distance analysis. Finally, 1754 CKD patients were included in the cross-sectional analysis. Klotho expression is known to decline in cases of acute kidney injury, regardless of whether it is caused by ischemia, infection, a toxin, or ureteral obstruction [14–16]. However, in the present study, we did not enroll patients with acute illness at the time of initial enrollment. Therefore, it is unlikely that initial klotho levels were influenced by acute illness. The study protocol was approved by the ethical committees of participating clinical centers, that is, by the Institutional Review Boards of Seoul National University Hospital (1104–089-359), Seoul National University Bundang Hospital (B-1106/129–008), Yonsei University Severance Hospital (4–2011-0163), Kangbuk Samsung Medical Center (2011–01-076), Seoul St. Mary’s Hospital (KC11OIMI0441), Gil Hospital (GIRBA2553), Eulji General Hospital (201105–01), Chonnam National University Hospital (CNUH-2011-092), and Pusan Paik Hospital (11–091) in 2011. All study subjects provided written informed consent. This study was performed in accordance with the principles of the Declaration of Helsinki.

### Clinical data collection and laboratory analyses

Laboratory values and demographic characteristics at enrollment were extracted from an electronic case-reporting form (http://www.phactax.org) developed by the assistance of the Division of Data Management at Seoul National University Medical Research Collaborating Center. Waist circumferences were measured midway between the lower part of the lowest rib and the upper iliac crest using a standardized protocol at clinics, as recommended by the World Health Organization (WHO) [17]. Brachial systolic blood pressure (SBP) and diastolic blood pressure (DBP) were obtained by averaging two measurements (separated by a minimum rest of 5 min in the sitting position) obtained using a calibrated oscillometric device (BP-203RV III; Omron Co., Kyoto, Japan). Serum creatinine levels were measured by an isotope dilution mass spectrometry (IDMS)-traceable method [18] at a central laboratory. Estimated glomerular filtration rate (eGFR) was calculated using the Chronic Kidney Disease Epidemiology Collaboration (CKD-EPI) creatinine equation [19]. Fasting blood glucose and triglyceride levels were measured in the hospital laboratories of participating center according to a standardized protocol. Overt proteinuria was defined as a 24-h urine protein result of > 500 mg/day. Serum α-klotho level was measured using an enzyme linked immunosorbent assay (ELISA) kit (Immuno-Biological Laboratories Co., Gunma, Japan) according to the manufacturer’s instructions [20]. The intra- and inter-assay coefficients of variation of this kit were 2.7–3.5% (klotho levels 186.64–2968.78 pg/mL) and 2.9–11.4% (klotho levels 165.47–2903.01 pg/mL), respectively. The data for intra- and inter-assay coefficients of variations were validated in our central laboratory by measurements of a serum control in 20 repeats on each ELISA plate. The intra- and inter-assay coefficients of variations were 0.57–1.78% and 3.01–6.12% (klotho levels 120.30–4468.50 pg/mL), respectively. The standard curve was shown to be linear up to 4000 pg/mL. C-terminal FGF23 was measured using a second generation human FGF23 ELISA kit (Immutopics, San Clemente, California, USA) according to the manufacturer’s instructions. The intra- and inter-assay coefficients of variation as informed by the manufacturer were 1.4–2.4% (FGF23 levels 33.7–302 RU/mL) and 2.4–4.7% (FGF23 levels 33.6–293 RU/mL), respectively.

### Definition of metabolic syndrome

MS was defined using the modified National Cholesterol Education Program Adult Treatment Panel (NCEP-ATP) III criteria according to American Heart Association/National Heart, Lung, and Blood Institute Scientific Statement, which adopted the Asian-Pacific waist circumference threshold by WHO. Subjects with three or more of the following 5 metabolic components were defined as having MS [2, 21]: 1) history of hypertension, elevated blood pressure (systolic blood pressure ≥ 130 mmHg, or diastolic blood pressure ≥ 85 mmHg), or taking anti-hypertensive medication; 2) history of diabetes mellitus, elevated fasting plasma glucose (≥100 mg/dL), or the use of anti-diabetes medication; 3) elevated triglycerides (≥150 mg/dL), or on drug treatment for elevated triglycerides; 4) reduced HDL cholesterol (< 40 mg/dL for men and < 50 mg/dL for women), or on drug treatment for reduced HDL cholesterol; and 5) elevated waist circumference (≥90 cm for men and ≥ 80 cm for women) according to the Asia-Pacific criteria [21, 22].

### Statistical analysis

Categorical variables were evaluated using the Chi-square test or Fisher’s exact test and presented as frequencies and percentages. Continuous variables were analyzed with independent sample *t*-tests or the Mann-Whitney U test. Results are presented as the mean ± standard deviation (SD) for normally distributed variables and the median (interquartile range [IQR]) for variables with skewed distributions. Log transformation was used to normalize klotho variability. We performed binominal logistic regression model analysis with adjustments (the Enter method), including variables that were significant in a univariate analysis or other clinically relevant variables to investigate the independent risk factors related to MS. To explore the association between MS and eGFR, or between MS and serum klotho considering the possible non-linearity, we adopted 3-knots restricted cubic splines with 95% confidence interval (CI) which described odds ratios (ORs) of MS according to the eGFR or klotho level. Knots were chosen using SAS LGTPHCURV9 Macro by Li et al. at the eGFR of 13.4, 42.7, and 106 ml/min/1.73m^2^; 257, 526, and 946 pg/mL for klotho, respectively. An eGFR of 60 ml/min/1.73m^2^, as a reference value of decreased renal function, and a serum klotho of 518 pg/mL (ROC [receiver operating characteristic] curve cut-off value) were taken as the reference point (OR = 1.00). The final model was adjusted for confounders. In subgroup analyses using an adjusted binominal logistic regression model (adjusted for age, sex, eGFR, overt proteinuria), we categorized patients according to age (aged < 50 vs. ≥50 years), sex, and CKD stages. *P-*values of < 0.05 were considered statistically significant. The SPSS statistical software (SPSS version 18.0, Chicago, IL, USA) and SAS (Version 9.4, SAS Institute Inc., Cary, NC, USA) were used for all descriptive and outcome analyses.

## Results

### Baseline clinical characteristics of subjects according to the presence of metabolic syndrome

**Fig. 1:**
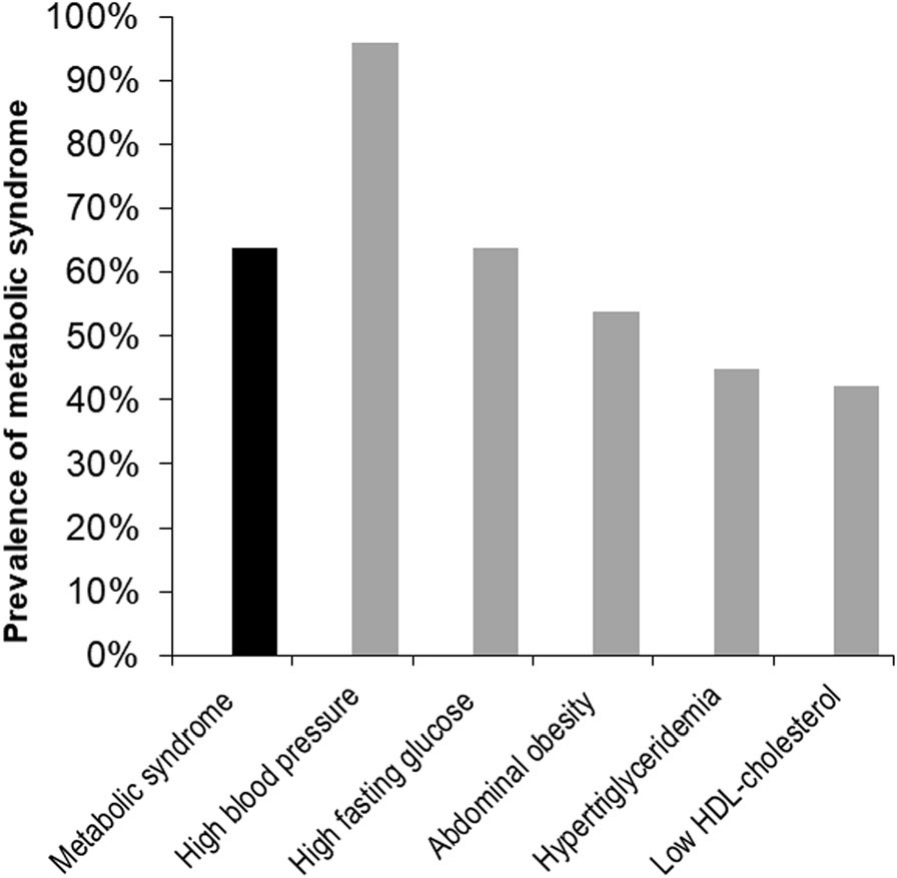
Prevalence of the metabolic syndrome and components of metabolic syndrome in the study subjects. Sixty four percent of patients had MS. Of the components of MS, high blood pressure (95.8%) was the most common, followed by high fasting glucose (63.7%) and abdominal obesity (53.8%). MS, metabolic syndrome; HDL, high-density lipoprotein

**Fig. 2:**
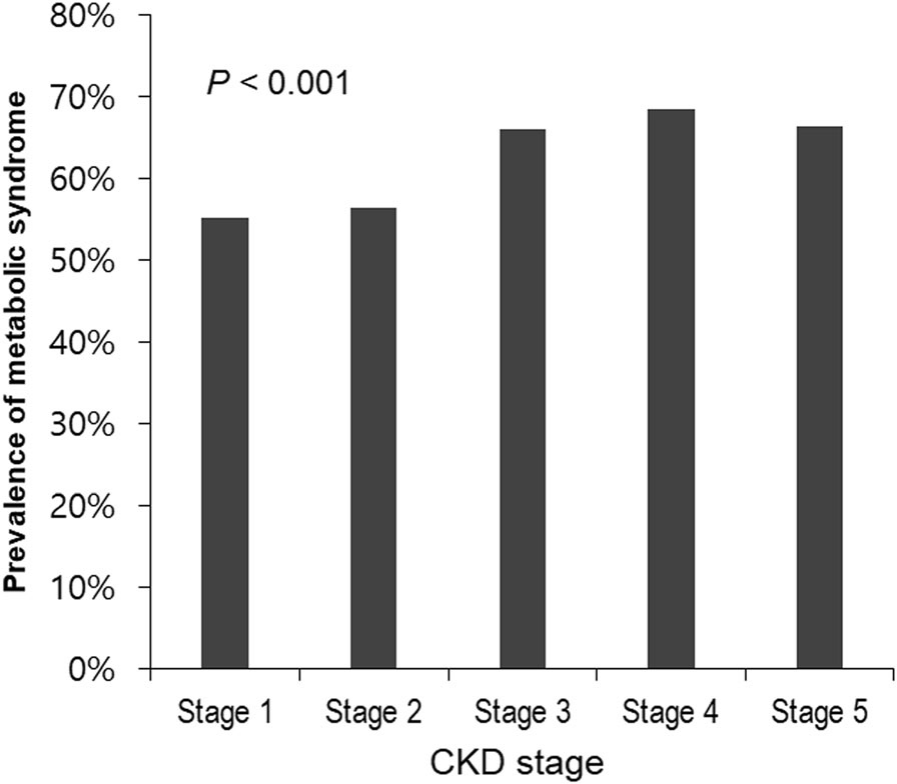
Prevalence of metabolic syndrome across CKD stages. The prevalence of MS was > 50% even for early stage CKD. The prevalence of MS was higher in advanced stages of CKD (*P* < 0.001). MS, metabolic syndrome

**Fig. 3:**
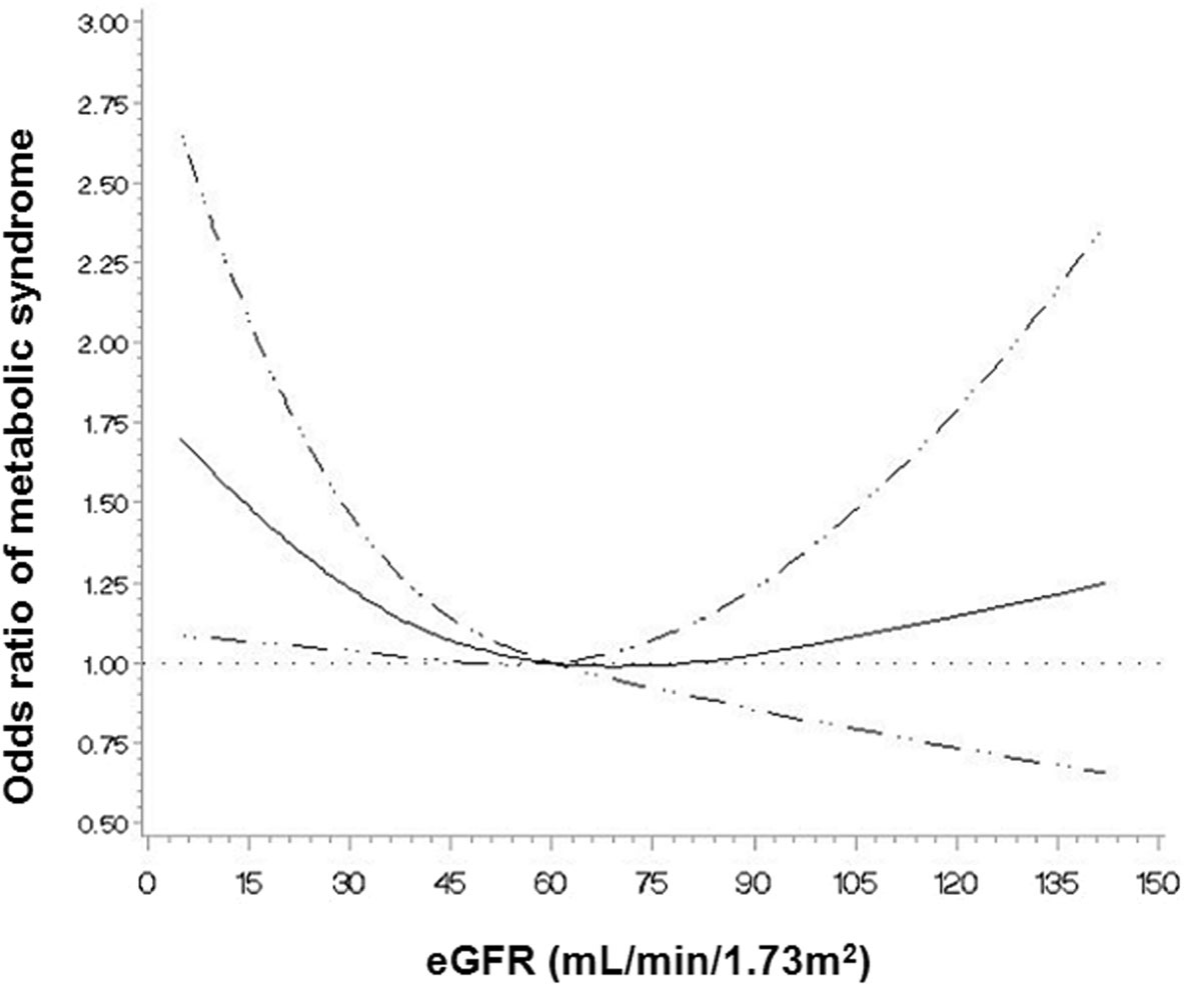
Multivariable-adjusted odds ratios of metabolic syndrome according to levels of estimated glomerular filtration rate. An eGFR of 60 ml/min/1.73m^2^, as a reference value of decreased renal function, was taken as the reference point (OR = 1.00). The adjusted OR of MS was significantly increased at eGFR levels of < 60 ml/min/1.73m^2^. The model was adjusted for age, sex, eGFR, and overt proteinuria. The solid line represents the multivariable-adjusted ORs of MS according to levels of eGFR. The dashed lines indicate 95% confidence intervals. eGFR, estimated glomerular filtration rate; OR, odds ratio; MS, metabolic syndrome

**Fig. 4:**
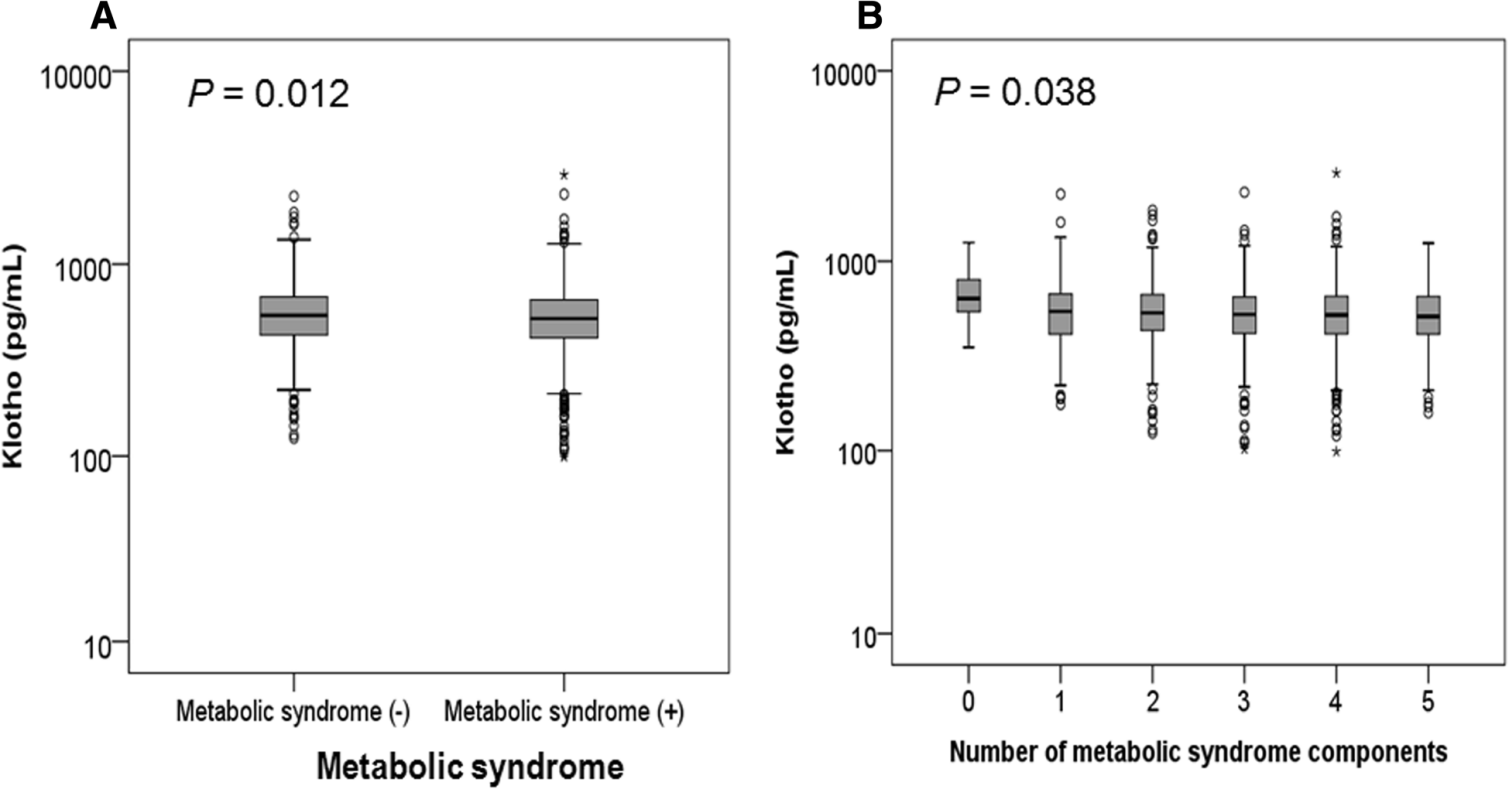
Serum klotho level according to metabolic syndrome and numbers of metabolic syndrome components. **a** Serum klotho was significantly lower in MS patients compared with patients without MS (median [interquartile range]; 521 pg/mL [413, 651] vs. 541 pg/mL [427, 676], respectively; *P* = 0.012). **b** Klotho levels tended to decrease as numbers of MS components increased (*P* = 0.038). MS, metabolic syndrome

The clinical characteristics of patients at enrollment are shown in Table 1. Mean age of the 1754 study subjects was 54.9 ± 12.1 years, and 1110 (63.3%) were male. Median serum klotho level was 527 pg/mL (IQR: 418–656 pg/mL). Among them, 1118 (63.7%) patients exhibited MS. The frequencies of the five MS component in the study subjects are shown in Fig. 1. High blood pressure (95.8%) was the most prevalent component, followed by high fasting glucose (63.7%) and abdominal obesity (53.8%). Figure 2 presents the prevalence of MS across CKD stages. The prevalence of MS was > 50% even for early stage CKD. The prevalence of MS was higher in advanced stages of CKD (*P* < 0.001, *P* for linear trend < 0.001). As show in Fig. 3, the adjusted OR (adjusted for age, sex, eGFR, and overt proteinuria) of MS was significantly increased at eGFR levels of < 60 ml/min/1.73m^2^. Subjects were divided into two groups according to the presence of MS. Patients with MS were older (*P* < 0.001, Table 1) and had higher blood pressure (*P* < 0.001), body mass index (*P* < 0.001), and waist circumference (*P* < 0.001). At enrollment, diabetes mellitus (DM) (*P* < 0.001), hypertension (*P* < 0.001), and preexisting CV disease (*P* < 0.001) were more prevalent among subjects with MS. Mean eGFR (*P* < 0.001) was lower and uric acid (*P* = 0.025) level was higher in MS patients. Serum klotho was significantly lower in MS patients compared with patients without MS (Median [IQR]; 521 pg/mL [413, 651] vs. 541 pg/mL [427, 676], respectively; *P* = 0.012; Fig. 4a). In addition, klotho levels tended to decrease as numbers of MS components increased (Fig. 4b; *P* = 0.038).

**Table 1:** The clinical characteristics of study subjects at enrollment with respect to the presence of metabolic syndrome

	Total (*N* = 1754)	Subjects without MS (*n* = 636)	Subjects with MS (*n* = 1118)	*P*–value
Age (years)	54.9 ± 12.1	52.3 ± 12.8	56.3 ± 11.4	< 0.001
Sex, male, n (%)	1110 (63.3)	387 (60.8)	723 (64.7)	0.111
SBP (mmHg)	128.5 ± 17.0	124.9 ± 16.4	130.6 ± 17.0	< 0.001
DBP (mmHg)	76.0 ± 11.2	75.5 ± 10.9	76.5 ± 11.4	0.059
BMI (kg/m^2^)	24.7 ± 3.4	22.9 ± 2.9	25.8 ± 3.2	< 0.001
Waist circumference (cm)	88.1 ± 9.8	81.4 ± 8.4	91.7 ± 8.5	< 0.001
DM, n (%)	700 (39.9)	110 (17.3)	590 (52.8)	< 0.001
Hypertension, n (%)	1719 (98.0)	606 (95.3)	1113 (99.6)	< 0.001
Preexisting CV disease, n (%)	298 (17.0)	72 (11.3)	226 (20.2)	< 0.001
CAD, n (%)	110 (6.3)	21 (3.3)	89 (8.0)	< 0.001
PVD, n (%)	76 (4.3)	22 (3.5)	54 (4.8)	0.175
Cerebrovascular disease, n (%)	110 (6.3)	24 (3.8)	86 (7.7)	0.001
HF, n (%)	28 (1.6)	9 (1.4)	19 (1.7)	0.648
Arrhythmia, n (%)	52 (3.0)	19 (3.0)	33 (3.0)	0.966
Cause of CKD, n (%)				< 0.001
GN, n (%)	746 (42.5)	387 (60.8)	359 (32.1)	
Diabetic nephropathy, n (%)	491 (28.0)	72 (11.3)	419 (37.5)	
Hypertension, n (%)	389 (22.2)	142 (22.3)	247 (22.1)	
Others, n (%)	128 (7.3)	35 (5.5)	93 (8.3)	
Laboratory findings				
eGFR (mL/min/1.73m^2^)	49.1 ± 28.6	53.1 ± 29.6	46.8 ± 27.9	< 0.001
Hemoglobin (g/dL)	12.7 ± 2.1	12.8 ± 2.0	12.7 ± 2.1	0.798
Albumin (g/dL)	4.1 ± 0.4	4.1 ± 0.4	4.1 ± 0.5	0.357
Uric acid (mg/dL)	7.2 ± 1.9	7.1 ± 1.9	7.3 ± 1.9	0.025
Creatinine (mg/dL)	1.9 ± 1.2	1.8 ± 1.1	2.0 ± 1.2	0.002
hsCRP, median, (IQR) (mg/dL)	0.07 (0.03, 0.17)	0.04 (0.02, 0.11)	0.08 (0.03, 0.21)	0.005
Fasting blood sugar (mg/dL)	113.9 ± 42.7	100.4 ± 28.5	121.5 ± 47.2	< 0.001
Total cholesterol (mg/dL)	173.4 ± 40.4	173.5 ± 36.2	173.3 ± 42.5	0.917
Triglyceride (mg/dL)	164.1 ± 103.1	106.3 ± 52.0	196.0 ± 110.2	< 0.001
LDL cholesterol (mg/dL)	95.0 ± 32.7	96.5 ± 30.1	95.7 ± 34.1	0.609
HDL cholesterol (mg/dL)	48.2 ± 15.5	56.5 ± 15.0	43.7 ± 13.8	< 0.001
Overt proteinuria^a^, n (%)	942 (61.0)	319 (58.3)	623 (62.5)	0.108
Medication, n (%)				
ACEi or ARB, n (%)	1521 (86.9)	526 (83.0)	995 (89.1)	< 0.001
Diuretics, n (%)	443 (25.3)	99 (15.6)	344 (25.3)	< 0.001
Statin, n (%)	998 (56.9)	323 (50.8)	675 (60.4)	< 0.001
Ca-based P binders, n (%)	161 (9.2)	67 (10.5)	94 (8.4)	0.179

^a^24-h urine protein > 500 mg/day

*MS* metabolic syndrome, *SBP* systolic blood pressure, *DBP* diastolic blood pressure, *BMI* body mass index, *DM* diabetes mellitus, *CV* cardiovascular, *CAD* coronary artery disease, *HF* heart failure, *PVD* peripheral vascular disease, *CKD* chronic kidney disease, *GN* glomerulonephritis, *eGFR* estimated glomerular filtration rate by CKD-EPI creatinine equation, *hsCRP* high sensitivity C-reactive protein, *IQR* interquartile range, *LDL* low-density lipoprotein, *HDL* high-density lipoprotein, *ACEi* angiotensin converting enzyme inhibitor, *ARB* angiotensin II receptor blocker, *Ca* calcium, *P* phosphorus

### Clinical characteristics of subjects stratified by serum klotho level

**Fig. 5:**
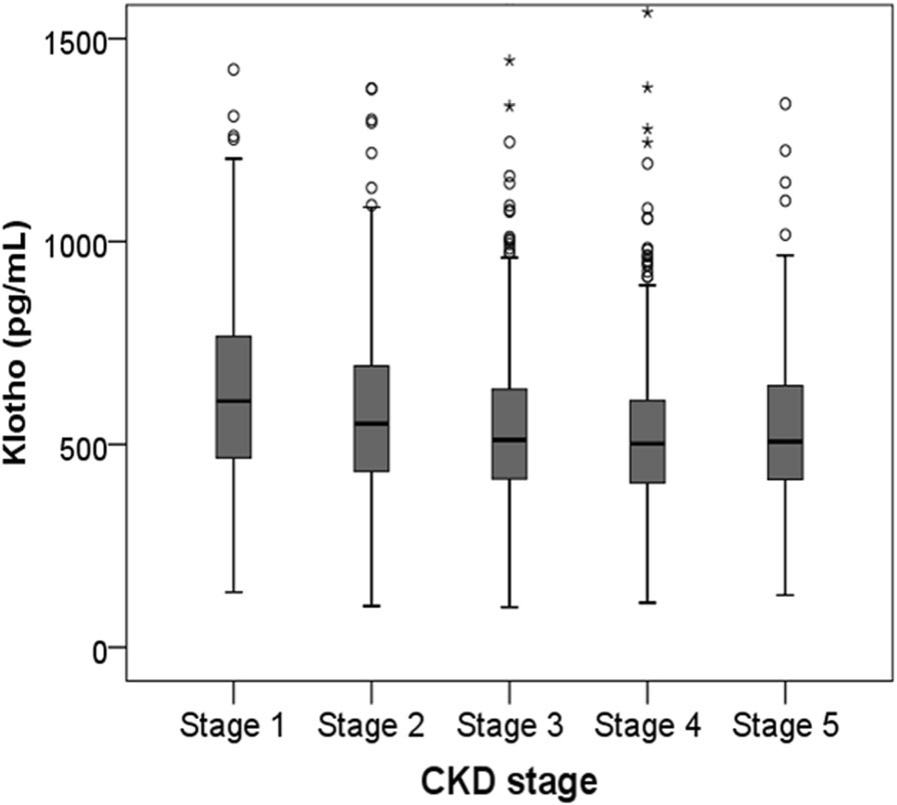
Serum klotho levels across CKD stages. Advanced CKD stages were associated with lower serum klotho levels (*P* < 0.001). CKD, chronic kidney disease

The clinical characteristics of patients dichotomized by serum klotho level (lower than median vs. equal to or higher than median) are shown in Table 2. Age age, sex, blood pressure, and underlying comorbidities were similar in these two groups. However, patients with a lower klotho level had a higher mean uric acid level (*P* < 0.001) and a higher C-reactive protein (CRP) level (*P* = 0.005). Mean eGFR (*P* < 0.001) and hemoglobin (*P* < 0.001) were lower in the low serum klotho level group. Serum klotho levels across CKD stages are shown in Fig. 5. Advanced CKD stages were associated with lower serum klotho levels (*P* < 0.001).

**Table 2:** Baseline characteristics of subjects stratified by serum klotho level

	Klotho groups		*P*–value
	Lower than median (*n* = 877) (99–526 pg/mL)	Equal to or higher than median (*n* = 877) (527–2909 pg/mL)	
Age (years)	55.3 ± 11.9	54.5 ± 12.3	0.152
Sex, male, n (%)	556 (63.4)	554 (63.2)	0.921
SBP (mmHg)	128.9 ± 16.7	128.2 ± 17.2	0.387
DBP (mmHg)	76.3 ± 11.5	76.0 ± 10.9	0.568
BMI (kg/m^2^)	24.9 ± 3.4	24.6 ± 3.4	0.060
Waist circumference (cm)	88.4 ± 9.8	87.8 ± 9.8	0.203
DM, n (%)	348 (39.7)	352(40.1)	0.845
Hypertension, n (%)	866 (98.7)	853 (97.3)	0.026
Preexisting CV disease, n (%)	144 (16.4)	154 (17.6)	0.525
CAD, n (%)	46 (5.2)	64 (7.3)	0.076
PVD, n (%)	40 (4.6)	36 (4.1)	0.639
Cerebrovascular disease, n (%)	50 (5.7)	60 (6.8)	0.325
HF, n (%)	11 (1.3)	17 (1.9)	0.253
Arrhythmia, n (%)	23 (2.6)	29 (3.3)	0.398
Cause of CKD, n (%)			0.012
GN, n (%)	358 (40.8)	388 (44.2)	
Diabetic nephropathy, n (%)	253 (28.8)	238 (27.1)	
Hypertension, n (%)	214 (24.4)	175 (20.0)	
Others, n (%)	52 (5.9)	73 (8.7)	
Laboratory findings			
eGFR (mL/min/1.73m^2^)	44.9 ± 26.0	53.6 ± 30.6	< 0.001
Hemoglobin (g/dL)	12.5 ± 2.0	13.0 ± 2.1	< 0.001
Albumin (g/dL)	4.1 ± 0.4	4.1 ± 0.5	0.953
Uric acid (mg/dL)	7.5 ± 1.9	7.0 ± 1.9	< 0.001
Creatinine (mg/dL)	2.0 ± 1.2	1.8 ± 1.1	< 0.001
hsCRP, median, (IQR) (mg/dL)	0.07 (0.03, 0.18)	0.06 (0.02, 0.15)	0.005
Fasting blood sugar (mg/dL)	110.9 ± 34.9	117.0 ± 49.1	0.003
Total cholesterol (mg/dL)	172.8 ± 39.7	174.0 ± 41.0	0.531
Triglyceride (mg/dL)	168.6 ± 107.0	159.7 ± 98.9	0.072
LDL cholesterol (mg/dL)	95.0 ± 32.3	97.0 ± 33.1	0.205
HDL cholesterol (mg/dL)	47.9 ± 15.9	48.6 ± 15.1	0.356
Overt proteinuria^a^, n (%)	475 (61.3)	467 (60.7)	0.821
Klotho, median, (IQR) (pg/mL)	418 (337, 475)	656 (583, 774)	< 0.001
Medication, n (%)			
ACEi or ARB, n (%)	773 (88.2)	748 (85.5)	0.088
Diuretics, n (%)	342 (39.0)	295 (33.6)	0.058
Statin, n (%)	514 (58.6)	484 (55.2)	0.309
Ca-based P binders, n (%)	88 (10.0)	73 (8.3)	0.396
Metabolic syndrome, n (%)	574 (65.5)	544 (62.0)	0.136

^a^24-hour urine protein > 500 mg/day

*MS* metabolic syndrome, *SBP* systolic blood pressure, *DBP* diastolic blood pressure, *BMI* body mass index, *DM* diabetes mellitus, *CV* cardiovascular, *CAD* coronary artery disease, *HF* heart failure, *PVD* peripheral vascular disease, *CKD* chronic kidney disease, *GN* glomerulonephritis, *eGFR* estimated glomerular filtration rate by CKD-EPI creatinine equation, *hsCRP* high sensitivity C-reactive protein, *IQR* interquartile range, *LDL* low-density lipoprotein, *HDL* high-density lipoprotein, *ACEi* angiotensin converting enzyme inhibitor, *ARB* angiotensin II receptor blocker, *Ca* calcium, *P* phosphorus

### Associations of metabolic syndrome and each metabolic syndrome component with serum klotho

**Fig. 6:**
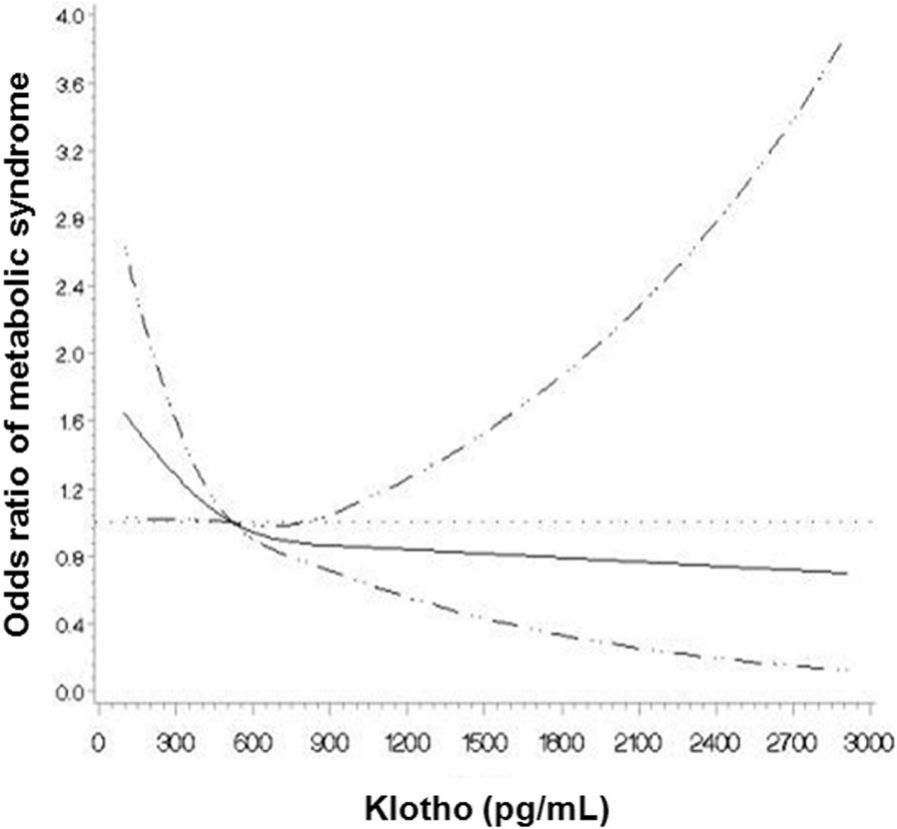
Multivariable-adjusted odds ratio of metabolic syndrome according to levels of serum klotho. A serum klotho level of 518 pg/mL (ROC curve cut-off value) was taken as the reference point (OR = 1.00). The adjusted OR of MS was significantly increased at serum klotho levels of < 518 pg/mL. The model was adjusted for age, sex, eGFR, and overt proteinuria. The solid line represents the multivariable-adjusted ORs of MS according to levels of eGFR. The dashed lines indicate 95% confidence intervals. Overt proteinuria, 24-hour urine protein > 500 mg/day; ROC, receiver operating characteristic; eGFR, estimated glomerular filtration rate; OR, odds ratio; MS, metabolic syndrome

In the univariate analysis, serum log klotho (OR, 0.40; 95% confidence interval [CI], 0.23–0.72; *P* = 0.002) and eGFR (OR, 0.99; 95% CI, 0.98–0.99; *P* < 0.001) were inversely associated with the presence of MS. On the other hand, age (OR, 1.03; 95% CI, 1.02–1.04; *P* < 0.001) was positively associated with MS. Binominal logistic regression analysis, adjusted for age, sex, eGFR, and overt proteinuria, showed that log klotho was independently associated with the presence of MS (adjusted OR, 0.44; 95% CI, 0.23–0.82; *P* = 0.010; model B in Table 3). As shown in Fig. 6, the adjusted OR of MS was significantly increased at serum klotho levels of < 518 pg/mL (the ROC cut-off value). Furthermore, adjusted for age, sex, eGFR, overt proteinuria, and drug information (ACEi or ARB, diuretics, statin), log klotho was still independently associated with the presence of MS (adjusted OR, 0.51; 95% CI, 0.27–0.98; *P* = 0.045).

**Table 3:** Multivariable logistic regression analysis presenting associations between log klotho and metabolic syndrome

Variable	Model A^a^	Model B^b^
	Adjusted OR (95% CI)	Adjusted OR (95% CI)
Log klotho	0.44 (0.25–0.80)^d^	0.44 (0.23–0.82)^d^
Age (per year)	1.03 (1.02–1.04)^d^	1.03 (1.02–1.04)^d^
Sex (male vs. female)	1.15 (0.94–1.41)	1.13 (0.91–1.40)
eGFR (per mL/min/1.73m^2^)		1.00 (0.99–1.00)
Overt proteinuria^c^		1.26 (1.01–1.58)^d^

*OR* Odds ratio, *CI* confidence interval, *eGFR* estimated glomerular filtration rate by CKD-EPI creatinine equation

^a^Model A adjusted for log klotho, age, and sex

^b^Model B adjusted for covariates in model A plus eGFR, and overt proteinuria

^c^24-hour urine protein > 500 mg/day

^d^significant association with MS (*P* < 0.05). *P* < 0.05 was considered significant

ORs of relations between each MS component and serum klotho are shown in Table 4. Only high blood pressure (adjusted OR, 0.05; 95% CI, 0.01–0.29; *P* = 0.001) and hypertriglyceridemia (adjusted OR, 0.48; 95% CI, 0.27–0.87; *P* = 0.016) were found to be independently associated with log klotho. High fasting glucose, abdominal obesity, and low HDL cholesterol were not significantly associated with serum klotho.

**Table 4:** Multivariable logistic regression analysis presenting associations between log klotho and each component of metabolic syndrome

Variable	Adjusted OR (95% CI)				
	High blood pressure	High fasting glucose	Abdominal obesity	Hypertriglyceridemia	Low HDL-cholesterol
Log klotho	0.05 (0.01–0.29)	1.32 (0.70–2.51)	0.68 (0.36–1.28)	0.48 (0.27–0.87)	0.71 (0.38–1.31)
*P*-value	0.001	0.389	0.235	0.016	0.271

Adjusted for covariates log klotho, age, sex, eGFR, and overt proteinuria

*OR* Odds ratio, *CI* confidence interval, *HDL* high-density lipoprotein, *eGFR* estimated glomerular filtration rate by CKD-EPI creatinine equation

### Subgroup analysis

We further analyzed the relationship between klotho and MS in several groups. Significant interaction was found in age group (aged < 50 vs. ≥50 years), suggesting that the association between klotho and MS was particularly evident in subjects aged < 50 years (adjusted OR, 0.18; 95% CI, 0.06–0.56; *P* = 0.003 vs. adjusted OR, 0.72; 95% CI, 0.33–1.57; *P* = 0.412 in aged ≥50 years). Klotho levels tended to decrease as age increased (Pearson’s correlation coefficient *r* = − 0.083, *P <* 0.001), but the value was not large. Klotho levels were not significantly different in aged < 50 and ≥ 50 years (Median [IQR]; 535 pg/mL [418, 668] vs. 525 pg/mL [418, 655], respectively; *P* = 0.465). There were no statistical interactions with sex group (interaction *P* = 0.605) and CKD stages (interaction *P* = 0.425) for the association between klotho and MS.

## Discussion

MS, a common clinical phenotype demonstrating as a combination of metabolic abnormalities including, hypertension, hyperglycemia, hypertriglyceridemia, decreased high-density lipoprotein cholesterol, and central obesity, is common in CKD [23]. In the present study, 63.7% of the 1754 CKD patients enrolled exhibited MS. The prevalence of MS exceeded 50% even in patients with early stage CKD and its prevalence was higher in advanced CKD stages. In the univariate analysis, serum klotho was significantly lower in patients with MS. After adjusting for various factors, such as, age, sex, eGFR, and overt proteinuria, serum klotho level remained an independent factor associated with MS in CKD.

In previous studies, genetic variants of *Klotho* increased the risk of MS which could be related to their susceptibility to high fasting glucose, high blood pressure, hypertriglyceridemia, and decreased HDL cholesterol [8, 12]. Klotho has a role in the regulation of vascular tone through homeostatic interplay between the renin-angiotensin system and nitric oxide [24], which might explain the association between klotho and blood pressure. Furthermore, the *Klotho* has been shown to be a target gene for PPARγ, a key transcription factor controlling lipid metabolism and insulin sensitivity [25]. Thus, we speculated these factors might play major roles in the association of klotho with glucose and lipid metabolism.

MS is a cluster of risk factors of diabetes and CV disease. Subjects with CKD present higher incidence of CV disease and higher CV mortality than the general population [26, 27]. Novel risk factors such as persistent inflammation, oxidative stress, and endothelial dysfunction can be linked to CV disease in CKD patients [28]. Fundamental manifestations of MS include insulin resistance and adipose tissue expansion, and the latter leads to oxidative stress and chronic inflammation, which aggravate insulin resistance [29–31]. Furthermore, these pro-inflammatory manifestations of MS might play roles in the pathogenesis of endothelial dysfunction and atherosclerosis [32]. *Klotho* is an anti-aging gene that extends life span when overexpressed and accelerates aging when disrupted [6]. Aging which reflects cellular senescence is closely associated with inflammatory reactions, oxidative damage, and endothelial dysfunction [33–35]. Klotho has also been shown to be an anti-inflammatory modulator [36, 37], to be closely associated with oxidative stress [38, 39]. Klotho plays a role in the protection against endothelial dysfunction [10]. The kidney is the principal organ responsible for the production of klotho, and CKD is known to be a klotho deficient state. Accordingly, the effects of klotho mentioned above suggest an association between klotho and MS beyond individual components of MS.

The present study is the first study, to our knowledge, to report the independent association between serum klotho level and MS per se especially in CKD patients. In previous studies, there were not many human studies, and most of studies were related to the association between genetic variants of *Klotho* and individual components of MS. In addition, few studies have been performed in patients with CKD. Furthermore, it was the strength of our study that we included a large-scale CKD cohort patients and obtained serum klotho level from a large number of our CKD patients. However, the study has several limitations that warrant consideration. First, because it was based on cross-sectional analysis, it could not demonstrate causality between klotho and MS. It could not provide detailed mechanistic links between klotho and MS. We just described the association between klotho and MS, not the causality. Second, serum klotho exhibits circadian variations [40]. They showed that midnight serum klotho level was an approximately 40% reduction with gradual return to near baseline values in the early morning hours. Serum klotho level decreased less than 20% compared with morning klotho level until 6 p.m. In the present study, blood samples were not obtained at any fixed time of the day. However, circadian variation of serum klotho may have been less in our study because blood sampling was done before 6 p.m.

## Conclusions

Serum klotho levels were found to be independently and inversely associated with the presence of MS. Further studies are warranted to elucidate the nature of the mechanistic link between klotho and MS in CKD patients.

## Abbreviations

ACEi: Angiotensin converting enzyme inhibitor
ARB: Angiotensin II receptor blocker
Ca: Calcium
CI: Confidence interval
CKD: Chronic kidney disease
CKD-EPI: Chronic kidney Disease Epidemiology Collaboration
CRP: C-reactive protein
CV: Cardiovascular
DBP: Diastolic blood pressure
DM: Diabetes mellitus
eGFR: Estimated glomerular filtration rate
ELISA: Enzyme-linked immunosorbent assay
HDL: High-density lipoprotein
IDMS: Isotope dilution mass spectrometry
IQR: Interquartile range
KNOW-CKD: KoreaN Cohort Study for Outcome in Patients With Chronic Kidney Disease
MS: Metabolic syndrome
NCEP-ATP: National Cholesterol Education Program Adult Treatment Panel
OR: Odds ratio
P: Phosphorus
ROC: Receiver operating characteristic
SBP: Systolic blood pressure
SD: Standard deviation
WHO: World Health Organization
